# Effectiveness of individualized counseling on the duration of exclusive breastfeeding: study protocol for a multicenter, randomized, parallel, and open clinical trial

**DOI:** 10.1186/s13063-023-07490-y

**Published:** 2023-07-15

**Authors:** Mariana Torreglosa Ruiz, Elisa da Conceição Rodrigues, Karine Emanuelle Peixoto Oliveira da Silva, Cynthya Viana de Resende, Michele Curcino Cavalcanti, Luciano Marques dos Santos, Monika Wernet, Ana Letícia Monteiro Gomes, Marialda Moreira Christoffel, Maria Beatriz Guimarães Raponi, Jéssica Aparecida da Silva, Jacqueline Faria de Oliveira, Divanice Contim, Ana Maria Linares

**Affiliations:** 1 Didactic-Scientific Department of Nursing in Hospital Care, Institute of Health Sciences, Federal University of Triangle Mineiro, Av. Getúlio Guaritá, 107, Uberaba, Minas Gerais, Minas Gerais 38025-440 Uberaba, Brazil; 2 Graduate/Posgraduate Program in Nursing, Anna Nery School of Nursing, Rua Afonso Cavalcanti, Cidade Nova 20211110, 275 Rio de Janeiro, Brazil; 3 Collegiate of the Nursing Course, State University of Feira de Santana. Avenida Transnordestina, Health Department, SN 44036-900 Feira de Santana, Bahia, Brazil; 4 Stricto Sensu Graduate Program in Health Care, Federal University of Triangle Mineiro, Av. Getúlio Guaritá, Av. Getúlio Guaritá, 107, Minas Gerais 38025-440 Uberaba, Brazil; 5 Stricto sensu Graduate Program in Nursing, Anna Nery School of Nursing, Rua Afonso Cavalcanti, 275, Cidade Nova, 20211110 Rio de Janeiro, Brazil; 6 Health Department, Collegiate of the Nursing Course, State University of Feira de Santana, Avenida Transnordestina, SN 44036-900 Feira de Santana, Bahia, Brazil; 7 Pos graduate Program in Nursing Federal, University of Sāo Carlos, Rodovia Washington Luis, km 235, 13565905 São Paulo, Brazil; 8 Graduate in Nursing, Anna Nery School of Nursing, Rua Afonso Cavalcanti, 275, 20211110 Rio de Janeiro, Rio de Janeiro, Brazil; 9 Institute of Nursing, Granja Dos Cavaleiros, UFRJ Multidisciplinary Center - Macaé, Federal University of Rio de Janeiro, Av. Aluizio da Silva Gomes, 50, Macaé 27930560 Rio de Janeiro, Brazil; 10 School of Medicine, Nursing Course, Federal University of Uberlândia, Avenida Pará, Bloco 2U, 1720, Umuarama, Minas Gerais 38400-902 Uberlândia, Brazil; 11 Multiprofessional Integrated Residency Program in Health - Integrated and Humanized Health Care, Federal University of Triangle Mineiro, Av. Getúlio Guaritá, Minas Gerais CEP, Av. Aluizio da Silva Gomes, 50, Macaé 38025-440 Rio de Janeiro, Brazil; 12 Clinic Hospital of Federal University of Triangle Mineiro, Av. Getúlio Guaritá, Minas Gerais CEP, Uberaba, 13038025-440 Brazil; 13 Didactic-Scientific Department of Nursing in Hospital Care, Institute of Health Sciences, Federal University of Triangle Mineiro, Av. Getúlio Guaritá, 107, 38025-440 Uberaba, Brazil; 14 University of Kentcky, USA, *27 Rose St, KY 40536 Lexington, USA

**Keywords:** Counseling, Breastfeeding, Rooming-in, Clinical trial, Nurses

## Abstract

**Background:**

Despite the benefits of breastfeeding, early weaning is a reality, so less than 50% of children worldwide and in Brazil are on exclusive breastfeeding in the sixth month of life. A strategy to counteract this scenario is breastfeeding counseling. This study aims to verify the effectiveness of individualized counseling by nurses trained in breastfeeding counseling, on the duration of exclusive breastfeeding, compared to standard care.

**Methods:**

Multicenter, randomized, parallel, and open clinical trial, with primiparous women aged over 18 years, hospitalized in rooming-in wards at participating centers and hemodynamically stable, aware, and oriented, who had a single-fetus pregnancy and gave birth, regardless of the type of delivery, with live child, gestational age of 37 to 42 weeks and birth weight greater than 2500 g. The women will be initially approached in rooming-in wards and, upon consent to participate in the study, will be allocated through randomization by blocks composed of eight participants in two groups: intervention and control. The randomization lists will be organized by a central without involvement with the study, which will manage the allocation groups and be prepared in the Randon® program. Women allocated to the intervention group will receive breastfeeding counseling by trained nurses, and those in the control group will receive standard care at the center participating in the study.

**Discussion:**

The results can contribute to breastfeeding by evidencing possible exclusivity and duration of the counseling trained nurses provide.

**Trial registration:**

REBEC RBR-4w9v5rq (UTN: U1111-1284–3559) (https://ensaiosclinicos.gov.br/rg/RBR-4w9v5rq). Posted on March 20, 2023.

**Supplementary Information:**

The online version contains supplementary material available at 10.1186/s13063-023-07490-y.

## Introduction

The World Health Organization defines breastfeeding counseling as support for mothers and newborns (NB) provided by health professionals, helping the woman and her family in decision-making and overcoming possible difficulties [1, 2]. It had its first description in 1993. It is based on dialogic interaction between counselors and women who breastfeed or intend to breastfeed. Thus, they aim to empower and support women in breastfeeding, respecting their reality and personal desires [1–3]. It is a horizontal, person-centered approach that goes beyond clinical management and guidelines for successful breastfeeding, meeting the principles of authenticity, positive feelings, and empathy, based on therapy centered on the needs of the client (counseling) by Carl Rogers and listening without judgment [1, 2].

To implement counseling, professionals need specific training, with a theoretical and practical workload ranging from 20 to 40 h, where listening and learning skills are worked on, in addition to increasing confidence and providing support to women who breastfeed or want to breastfeed. Although counseling is considered a light technology to support breastfeeding, not all breastfeeding support is provided through this approach [3].

Thus, despite the description of positive results with the implementation and strategy dating from the 1990s, it is observed that there still needs to be a description of protocols for this intervention, especially concerning its application during the hospitalization of the dyad in rooming-in wards. It is important to emphasize that it differs from the process of health education and clinical management focused on breastfeeding, justifying the originality and the need to carry out this study.

The need to test interventions to promote breastfeeding is reinforced since the perception of support from mothers during hospitalization significantly predicts exclusive breastfeeding [4]. Thus, providing an evidence-based intervention to support hospitalized mothers is critical [4].

A systematic review study with meta-analysis pointed out the positive effect of the intervention in reducing weaning before the sixth month (RR = 0.91) and before 6 weeks (RR = 0.87). However, it is considered moderate-quality evidence due to the significant heterogeneity of the analyzed studies [3].

The literature points out as results of counseling: increased rates of exclusive breastfeeding [5–8]; and an increase in the duration of breastfeeding, including mixed breastfeeding [9, 10] and in the exclusive and mixed forms [11]. Some studies, however, did not identify differences with its implementation [12, 13], but the great sample heterogeneity is highlighted, related to the number of maternal pregnancies; gestational age; birth weight, and, as pointed out, the absence of specific protocols for its performance.

In addition, no clinical, randomized, multicenter Brazilian trial has been identified that studied the effectiveness of individualized counseling carried out by nurses trained in counseling during the duration of exclusive breastfeeding. Therefore, this research project will test the following alternative hypothesis: individualized counseling provided by trained nurses is effective in the duration of exclusive breastfeeding compared to standard care. As a null hypothesis: individualized counseling performed by trained nurses is ineffective in the duration of exclusive breastfeeding, compared to standard care.

Therefore, this study aims to verify the effectiveness of individualized counseling performed by trained nurses on the duration of exclusive breastfeeding and on occurrence of breast complications compared to standard care.

## Methods

This clinical trial has the following specific objectives:

1. Compare the percentage of occurrence of nipple-areolar lesions in the first and second weeks of life for the intervention and control groups;
2. Compare breast complications in the first 2 weeks based on mother reports and compare results between groups;
3. Compare the percentage of exclusive breastfeeding in the first, fourth, and sixth month in both groups;
4. Evaluate postpartum depression scores at different times (1, 4, and 6 months) and compare the occurrence between the two groups;
5. Compare the rate of hospitalizations and occurrence of childhood illnesses at 2, 4, and 6 months of life, according to the type of breastfeeding and between groups;
6. Compare the percentage of weaning between groups;
7. Evaluate the care complexity of the dyads (minimal, intermediate, semi-intensive, and intensive care) participating in the study, based on a validated scale, and
8. Present the performance scores of the nursing mother and the baby during breastfeeding during hospitalization in the rooming-in wards, in the two study groups, based on a validated scale (LACTH).

### Study design

This is a multicenter, randomized, superiority, parallel, and open clinical trial guided by the international recommendations of the Consolidated Standards of Reporting Trials [14].

The randomized clinical trial is considered the gold standard for evaluating health interventions, establishing a causal relationship, and evaluating the effectiveness of the intervention [15].

This is a superiority study, because it will determine whether the investigational intervention is superior to the comparator; parallel study, as each group of participants will be exposed to only one intervention studied (intervention or control); random, as participants will be allocated between groups, and each individual has the same probability of being selected for one group or another [16, 17]; and open, as there will be no masking of the intervention (participants and researchers) [18–21], because it would be impossible to blind this intervention for women and nurses. However, data analysis will be carried out by researchers not directly involved in data collection.

This clinical trial protocol was designed following the Standard Protocol Items: Recommendations for Interventional Trials (SPIRIT) [22] as evidenced in the checklist (see Additional File 1).

### Study location

The study will be carried out in the rooming-in wards of the hospitals: Clinics of the Federal University of Triangle Mineiro (HC-UFTM), in Uberaba (MG); Inacia Pinto dos Santos (HIPS), in Feira de Santana (BA) and at the Maternity School of the Federal University of Rio de Janeiro, in Rio de Janeiro (RJ).

In context, the Clinics of the Federal University of Triangle Mineiro (HC-UFTM) recorded 1259 deliveries in 2020. Reference for the resolution of high-risk pregnancies, infectious diseases in the pregnancy-puerperal cycle, for patients assisted in pathological prenatal care in municipalities in the Southern Triangle of Minas Gerais, and for pregnancies at usual risk located in the region of the institution and in all cities in the Southern Triangle of Minas Gerais that do not have a hospital. It is a public hospital with 100% of its services linked to the Unified Health System (SUS). It has 12 rooming-in beds and does not have the title of Baby-Friendly Hospital.

The Hospital Inacia Pinto dos Santos (HIPS) is characterized by being the first hospital in Bahia to receive the title of Baby-Friendly Hospital and being a reference in caring for women and newborns at normal risk. A public teaching hospital with three rooming-in units recorded 7501 deliveries in 2022 and has an installed capacity of 95 beds.

At the Maternity School of the Federal University of Rio de Janeiro, in 2020, 1607 births were recorded. Also characterized by public assistance and as a teaching hospital, its AC has nine wards totaling 45 active beds. Pregnant women of normal risk and those who need treatment for intercurrent illnesses or those related to pregnancy are assisted. The institution received the title of Baby-Friendly Hospital in December 2020.

### Inclusion and exclusion criteria for participants

Will be included in the study, primiparous, aged over 18 years; who were pregnant with a single fetus, with live birth, gestational age of 37 to 42 weeks, birth weight greater than 2500 g, regardless of the type of delivery; who are hemodynamically stable, conscious, and oriented and admitted to the rooming-in wards, of the participating centers, at the time of allocation for the study.

Postpartum women and newborns with contraindications for breastfeeding (HIV positive; HTLV 1 and 2 positive or neoplastic treatment with chemotherapy) will not be included; infants with malformations that prevent or make breastfeeding difficult or with changes in breastfeeding mechanics (lingual frenulum); puerperal women whose infants were immediately separated after clamping the umbilical cord at birth due to maternal-neonatal complications, in which one or both were hospitalized in critical units; postpartum women transferred from other institutions or who have already been discharged (rehospitalization), postpartum women using illicit drugs, postpartum women with intellectual or sensory disabilities with a medical diagnosis. These criteria contemplate the feasibility of the intervention and the necessary follow-up to the clinical trial.

Women who are not followed up in the first month, after three unsuccessful attempts at contact and at least one contact with a family member at different times, will also be excluded if malformations or abnormalities in the mechanics of breastfeeding or changes in the mother–child bond are detected at the time of allocation.

### Randomization, concealment of allocation, and masking

The sample will be probabilistic and calculated after conducting a pilot test with 60 postpartum women (20 in each center). A significance level of 5% and a statistical power of 95% will be considered. The postpartum women will be recruited by the research team, duly qualified for this action, and allocated through randomization by blocks composed of eight participants in two groups: intervention and control.

The randomization lists of each research center will be organized by a central of randomization, which will manage the allocation groups. This list will be prepared using the Randon® program. The researchers will contact the members of the randomization center to consult the order of inclusion of postpartum women in the research and their allocation group. This center will carry out allocation concealment, which will not be directly involved in the study data collection. There will be no blinding of participants and researchers involved in the collection.

### Intervention

The non-pharmacological intervention to be tested will be breastfeeding counseling during the dyad hospitalization in the rooming-in wards, developed by a nurse trained in breastfeeding counseling. The control group will receive institutional assistance. Breastfeeding actions, in Brazil, are not systematized but focused on clinical management and health education, prioritizing binomials at high risk for weaning [23].

### Experimental group

In the intervention group, counseling will be carried out by a team of nurses qualified for breastfeeding management and counseling.

Women will be encouraged to answer the following open questions: How are you breastfeeding? Moreover, if she has doubts or concerns, they will be explored with: Tell me about it… Next, the following guiding question will be used: How are you feeling breastfeeding? Furthermore, in the end, you will be asked: What led you to breastfeed? The validated protocol for counseling on breastfeeding during hospitalization in rooming-in wards will be used for all responses.

Postpartum women allocated to this group will be approached using counseling, as described above. The researcher will collect the history of breastfeeding with the counseling approach and observe the breastfeeding. During breastfeeding, the LATCH [24] scale items will be observed—latching on, swallowing, type of nipple, comfort, and positioning. Afterward, the researcher will interview the puerperal woman and fill in sociodemographic, clinical, and obstetric data.

At the end of the first counseling session, the researcher will fill in the items of the scale adapted from the observation and evaluation form for rooming-in wards—Fantinelli’s scale [25], which measures the care complexity of the dyad.

Counseling will be offered twice a day until hospital discharge in the morning and afternoon, and the start and end times of the intervention will be recorded to determine the duration.

At the end of the intervention, the women will be invited to watch a videoscribe with the theme “Breastfeeding is better,” produced by the study group, in which the advantages of breastfeeding for the mother and newborn are discussed and guidance is given to seek help in case of breast complications. The video has three distinct versions, with the voices of the researchers of each center and a version in Brazilian Sign Language. The version adapted to the participant’s location will be used. The Brazilian Sign Language version was created for inclusion and health education purposes for these women but will not be used in this study.

The intervention (implementing individualized counseling on the duration of exclusive breastfeeding) will not require alteration to usual care pathways, including use of any medication for postpartum woman or neonates and these will continue for both trial arms.

After hospital discharge, telephone contacts will be made through the number provided by the participant to collect the outcomes. To ensure that there is no loss of contact, the researcher will request the contact of the postpartum woman and a family member at the time of allocation. Contact will be made by the researcher who collected the data with the postpartum women included to maintain the link with the postpartum women and reduce sample losses. Contacts will be made: in the first and second weeks of life, with 1, 4, and 6 months of the child’s life to collect the outcomes.

It should be noted that all researchers at the participating centers are not part of the care teams at the units. All were trained to intervene with the Clinical Management of Breastfeeding course (60 h) and Counseling Workshop (16 h), including practical activities, totaling 76 h of training.

### Control group

The control group will receive standard institutional care, that is, the assistance offered by a professional, from the institution itself, without intervention by the researchers.

In these cases, the researcher responsible for data collection will conduct the interview and non-participant observation of at least one feeding without applying any intervention. The same instruments used in evaluating the intervention group will be applied. At the end of the interview, participants will also be invited to watch the videoscribe produced for the study.

Telephone contacts will be made, as well as in the intervention group, in the first and second weeks of life, with 1, 4, and 6 months of the child’s life to collect the outcomes.

### Criteria for discontinuing the intervention

Interim analyses will be performed every 60 days during data collection and presented to a clinical trial safety monitoring committee to assess the continuity of data collection, given the findings of adverse events or statistical differences between the groups surveyed concerning primary and secondary outcomes. The data analysis will be of the type by the finalization of the protocol, denominated explanatory.

This committee is highly recommended for RCTs being utilized by the FDA and NIH. It is suggested that in its minimum composition, it has a specialist in the subject, a specialist in RCT, a specialist in statistics/epidemiological studies, a representative of the Ethics Committee, and a representative of the users (who are in the same condition as the study participants) [26]. The proposed committee for this study will consist of five representatives, two specialists in the subject and in conducting RCTs, one specialist in epidemiological studies and statistics, a representative of the Ethics Committee, and a representative of the users. The participants of this committee will not be involved in the study team and have indicated that they are aware of this condition.

The study must be stopped if there is definitive evidence, during its conduction, of the benefit or lack of benefit of the intervention, which the specific Monitoring Committee will evaluate for this randomized clinical trial [26].

### Data collection instruments

The data collection instrument developed specifically for this study was submitted to content validation by eight experts with a doctorate and relevant experience in Breastfeeding Counseling, teaching, and evidence-based research.

The baseline instrument contains three parts, namely: Part I—sociodemographic variables: age; self-declared race marital status, and, if living with a partner, age, and level of education; schooling, family income, occupation, maternity leave, paternity leave, origin and presence of a companion at the time of the interview; Part II—clinical variables: smoking, coffee drinking, health problems, and medication use; Part III—obstetric variables: prenatal care and the number of consultations; guidance on breastfeeding during prenatal care and sources, including specification in case of professional guidance. Information about the delivery/birth will be collected, such as date of delivery, type of delivery, if the puerperal woman had a companion during labor and delivery; if there was early contact with the newborn, and if the mother started breastfeeding in the first hour of life. About the NB, weight, height, gestational age, and type of breastfeeding data will be collected at the time of hospital discharge.

The information will be collected through interviews and medical records.

Women allocated to the intervention group will be encouraged to answer the following open questions: How are you breastfeeding? Moreover, if she has doubts or concerns, they will be explored with: Tell me about it… Next, the following guiding question will be used: How are you feeling breastfeeding? Furthermore, in the end, you will be asked: What led you to breastfeed? The validated protocol for counseling on breastfeeding during hospitalization in rooming-in wards will be used for all responses.

For evaluation based on observation of breastfeeding, the LACTH [24] scale will be used—attachment, swallowing, type of nipple, comfort, and positioning, applied to both groups, based on observation of at least one breastfeeding.

A nipple-areolar evaluation will be performed, regarding skin color and type of nipple [27]. In case of nipple-areolar lesions detected by visual inspection, the Nipple-Areolar Lesions Classification Instrument [28] and the Nipple Trauma Score [29] will be applied.

The presence of breast or nipple pain will also be evaluated based on applying the Visual Analog Pain Scale [30, 31], and, in the case of pain, pain characteristics [32]. At the end of the interview, women from both groups will be invited to watch the videoscribe produced.

The researcher will record the scale items adapted from the observation and evaluation form for the rooming-in wards—Fantinelli’s scale, which allows for assessing the care complexity of the dyad [25].

In the first and second weeks of the newborn’s life, as well as in the first, fourth, and sixth months, three attempts to contact the postpartum woman by telephone will be made from the number provided in the initial contact, at different times, and at least one attempt to contact with familiar.

In the first and second weeks, data on the type of breastfeeding at the time will be collected; introduction of artificial milk, nipple-areolar lesions or pain, breast engorgement, use of pacifiers, professional search/support for breastfeeding issues, weaning, follow-up of childcare, and neonatal intercurrences. Interventions will not be applied to both groups.

From the first month, the Edinburgh Postpartum Depression Scale will be applied [33, 34]. Hospitalizations and childhood illnesses will also be questioned at 2, 4, and 6 months of the child’s life. The same questionnaire will be used for the following months until the conclusion of the protocol in the sixth month.

### Recruitment and data collection procedures

The researcher, daily, will go to the nurses responsible for the ward and locate the census of hospitalized puerperal women and identify the primiparous women who delivered at term and the bed in which they are hospitalized. The following information will be checked in the medical records: HIV and HTLV 1 and 2 serological test results; information on neoplastic diseases currently being treated with chemotherapy; neonatal malformations; diagnosis of neonatal ankyloglossia; description of separation at birth (hospitalization in critical units—women or newborn); readmission due to maternal or neonatal complications; illicit drug users; postpartum women with a medical diagnosis of intellectual difficulty or sensory deficit. If it has any of the present criteria, it will not be included in the list for randomization. If there are no criteria for non-inclusion, randomization will be performed. The gestational age and birth weight of neonates will also be checked.

Once the criteria are met, the researcher will go to the ward, explain the study’s objectives to the selected postpartum women and read the Informed Consent Form (ICF). When deciding positively to participate, the postpartum woman must sign the ICF in two copies, receiving a copy and another that the researchers will keep. The researcher will contact the randomization center, and eligible postpartum women will be randomized to the control or intervention group as directed by the center.

In the control group, the interview, evaluation of the breasts, and at least one feeding will be performed using the abovementioned instruments. At the end (zero time), the researcher will reinforce the telephone contacts and offer the videoscribe visualization. Unfilled information can be extracted from the medical records, and the care complexity scale will be filled in.

In the intervention group, the postpartum woman will be approached with the strategy of counseling, breast evaluation, and at least one breastfeeding and interview according to a validated protocol. Ultimately, the researcher will reinforce the subsequent contacts, which will succeed in the allocation, interspersed in the morning and afternoon, depending on time zero, until hospital discharge. In the follow-up at the hospital, an approach with a counseling strategy will be carried out, identifying doubts and potential needs. In the end, the videoscribe will be offered and reinforced about subsequent telephone contacts for follow-up and their importance.

To characterize the sampling, the researcher will complete a daily list of eligible postpartum women, included or not included, and reasons for non-inclusion.

If the researcher detects abnormalities in the mechanics of breastfeeding or changes in the mother–child bond, the care team members will be notified, and the participant must be excluded.

Follow-up interviews will be done through phone calls. The researcher will make three telephone contact attempts at different times of the day: between 12:30 pm and 1:30 pm, from 4:00 pm to 5:00 pm, or at another preferred time indicated by the participant. If the attempt is unsuccessful, the researcher will try to contact the number provided by the family member. If accepted, the researcher will apply the questionnaire, thank them, and inform a new contact within a week. Contact will be made by the researcher who collected the data with the postpartum women included to maintain the link with the postpartum women and reduce sample losses.

In the second week, the same procedures will be carried out, but at the end, the researcher will signal a new contact in 2 weeks; in the first month, he will inform that he will return in 3 months, and on the fourth month, he will inform that the next call will be in 2 months. At the end of the protocol, the researcher will close the research and thank the participant.

Postpartum women who are not followed up in the first 15 days, after three unsuccessful contact attempts and at least one contact with a family member at different times, will be excluded.

All data will be completed in Google Forms® electronic forms on tablets purchased for this purpose. Only researchers will have access to the electronic database, collection instruments, and consent forms.

**Fig. 1:**
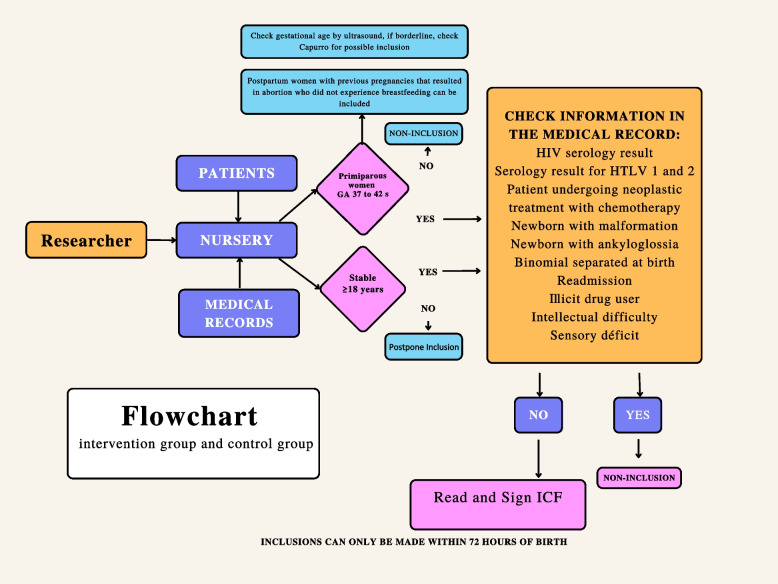
Flowchart for inclusion of participants in the study

**Fig. 2:**
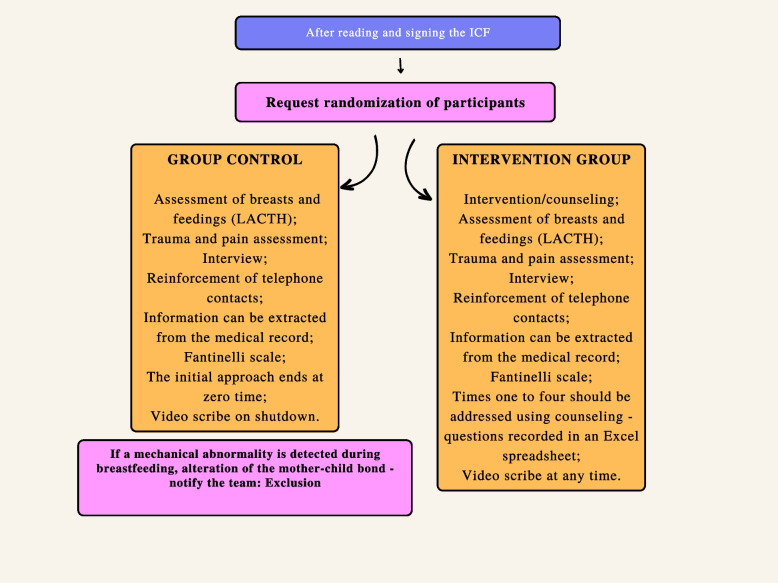
Randomization flowchart and collection procedures for control and intervention groups

**Fig. 3:**
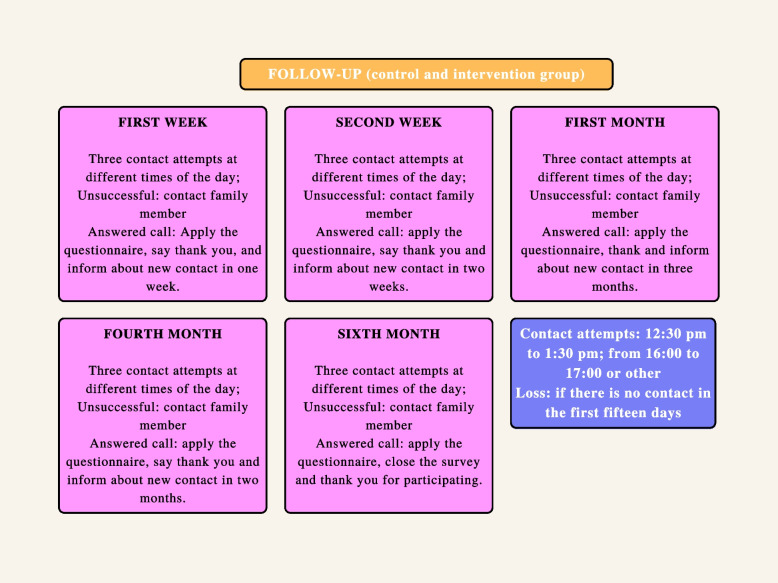
Follow-up flowchart for the control and intervention groups after hospital discharge

**Fig. 4:**
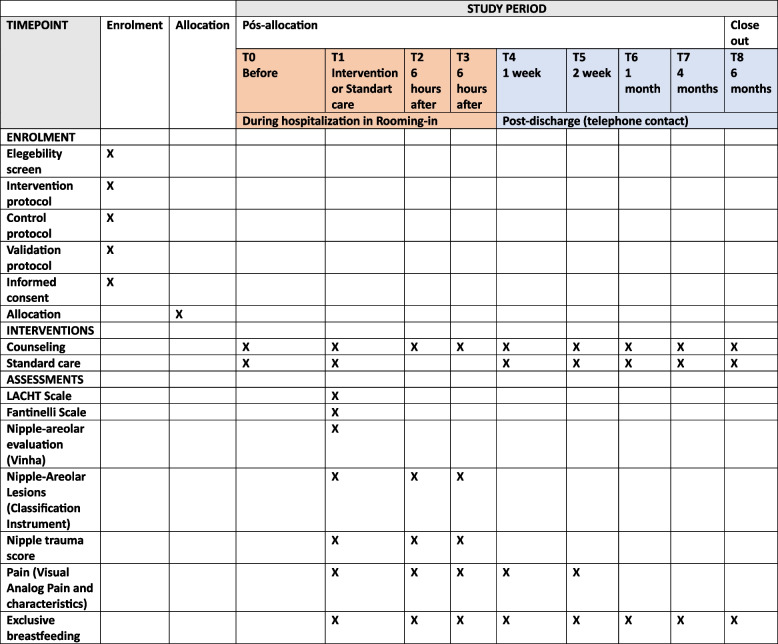
Standard Protocol Items: Recommendations for Interventional Trials (SPIRIT) flowchart

The flowchart for the inclusion of research participants is shown in Fig. 1, the randomization and data collection procedure and data steps are summarized in Fig. 2, and the follow-up after hospital discharge is described in Fig. 3. The flowchart for the research participants is shown in Fig. 4.

### Safety protocols

All biosecurity protocols will be strictly followed, and the data collection team will use all necessary personal protective equipment.

As for adverse events, their occurrence is not predictable in this research, given the nature of the intervention. However, in case of any complication or adverse event for the participants, the information will be registered, and the Research Ethics Committee will be immediately communicated.

It should be noted that the study will be stopped if there is definitive evidence, during its conduction, of the benefit or absence of benefit from the intervention, which the specific Monitoring Committee will evaluate for this randomized clinical trial.

### Evaluated outcomes

#### Primary outcome

- Duration of exclusive breastfeeding: the child who is exclusively breastfed, without the introduction of water, tea, liquids, milk supplementation, or food introduction, as recommended by the World Health Organization [35].

### Secondary outcomes

- Nipple-areolar lesions: lesion(s) in the nipple-areolar region(s) with or without disruption of the skin barrier.
- Nipple or breast pain: verbal report of pain, describing its characteristics, associated with breastfeeding.
- Breast complications: occurrence of engorgement or mastitis.
- Exclusive breastfeeding in the first, fourth, and sixth months of life: exclusive breastfeeding in this period.
- Postpartum depression scores: measured by the Edinburgh Scale, indicated for tracking signs indicative of puerperal depression, which should be applied after the persistence of symptoms of puerperal blues, that is, with continuity of symptoms after 1 month and consistent with the birth.
- Hospitalizations and occurrence of illnesses in childhood: based on the mothers’ report to the telephone contact.
- Weaning: interruption of breastfeeding, considered early, when it occurs before 4 months of the child’s life.
- Care complexity of the dyads: classification into minimal, intermediate, semi-intensive, and intensive care, based on the care required by postpartum woman and newborn.
- Performance of the postpartum women and newborn during breastfeeding: based on the validated scale (LACTH) application.

### Statistical methods

#### Sample size

The sample size will be calculated by a statistician with no clinical involvement in the research and obtained after carrying out the pilot study.

Previously, a pilot study will be conducted with 60 participants being recruited, 20 participants from each center, 10 allocated in the intervention group, and 10 in the control group for each center. To achieve a high confidence level for a pilot study, a minimum sample of 50 research subjects is recommended [36]. A significance level of 5% and a statistical power of 95% will be considered, and the sample calculation will be performed after analyzing the pilot study, as well as follow-up losses will be considered, using the OpenEpi version 3.01 program.

### Statistical analysis

Data will be entered into a Google Forms® form, saved as Microsoft Excel® spreadsheets, and imported into the statistical program Social Package for the Social Sciences (SPSS) version 23.0 and analyzed in this same program and OpenEpi version 3.01. The data analysis will be of the type by the finalization of the protocol, denominated explanatory.

In characterizing the allocation groups in the research, measures of absolute and relative frequencies will be used for the categorical variables. Numerical variables will be summarized using measures of central tendency (mean or median) and dispersion (standard deviation, minimum, and maximum).

To verify the relationship between the exposure variable and the outcomes, Pearson’s *χ*^2^ and Fisher’s exact tests will be used for categorical variables and Student’s *T* test or Mann–Whitney, for numeric variables, considering a significance level of 5%, relative risks and their 95% confidence intervals. From the initial analysis, analyses of subgroups and adjusted analyses will be used.

### Ethical aspects

The study will follow the ethical principles of the Declaration of Helsinki and Resolution No. 466, of December 12, 2012, of the National Council of Health Council, Brazil, which deals with regulation Guidelines and standards for research involving human subjects [37].

The project was submitted for the approval of the Women’s Health Unit of the Clinics of the Federal University of Triangle Mineiro, the clinical board of the Maternity School of the Federal University of Rio de Janeiro, and the Permanent Education Center of the Hospital Inacia Pinto dos Santos.

After collecting the consent at the study centers, it was registered at the Research Management Unit of the Clinics of the Federal University of Triangle Mineiro (GEP-HC-UFTM) and the Rede Pesquisa System (of university hospitals). After registration, it was registered on the Plataforma Brasil CAAE 61321122.3.1001.8667, with the registration of all co-participating institutions and centers.

The project was approved by the Ethics Committee of the Clinics of the Federal University of Triangle Mineiro (HC-UFTM), opinion n° 5,627,159 of September 6, 2022, and the Maternity School Committee of the Federal University of Rio de Janeiro, opinion n° 5,656,072 of September 21, 2022.

The translated and validated LATCH scale [24] was kindly provided by the study authors, and the Fantinelli scale [25], was also validated and provided for use by the author to assess the care complexity of the dyads.

This research was registered on the Brazilian Clinical Trials Registry Platform (ReBEC) under RBR-4w9v5rq (UTN: U1111-1284–3559).

Participants will receive complete information about the research, such as objectives, procedures, probability of participating in the control or intervention group, risks, and benefits, as well as clarifications about voluntary participation and the possibility of withdrawing from the study at any time without prejudice to treatment. After reading the clarification form, participants will sign two copies of an informed consent form, one for the participant and one for the researcher.

To minimize the risk of loss of data confidentiality, participants will be identified in the survey by codes. At no time will personal data or any information that may identify participants be disclosed.

All documents generated from the research, such as data collection instruments and forms, will be kept in a safe place with restricted access. After the conclusion of the clinical trial, they will remain on file for 5 years, being subsequently erased, under the responsibility of the coordinating researcher.

It should be noted that any need and modifications to the study protocol will be documented as a protocol amendment and must be formally communicated to the Research Ethics Committee, the Brazilian Registry of Clinical Trials platform, and the journal in which the protocol will be published.

### Access to data

The datasets analyzed during the current study and statistical code are available from the corresponding author on reasonable request, as is the full protocol.

### Ancillary and post-trial care

This care will not be necessary in the study. In the event of damage or loss to any participant as a result of their participation in the research, due assistance, and compensation, determined by the institution’s Research Ethics Committee, will be made available.

### Dissemination of results

When the study is completed, the data will be de-identified prior to data sharing. The results of the study will be disseminated to the academic and professional community, through release of public access to the dissertations, thesis, publication of scientific articles, and presentation of papers at scientific events.

## Discussion

This is a protocol for a randomized, controlled, multicenter, parallel, and open clinical trial, which aims to evaluate the effect of counseling on breastfeeding developed by nurses during the hospitalization of the dyad in rooming-in wards on the duration of exclusive breastfeeding.

Worldwide, 80% of newborns receive breast milk at some point in their lives [38]; 48% start breastfeeding in the first hour of life, but this rate drops to 44% when evaluating the exclusive form up to the sixth month of life [35, 38]. In Brazil, the prevalence of exclusive breastfeeding up to 6 months of age is 45.8% [39].

Thus, this research is justified because of the high rates of weaning in Brazilian children and the world; previous studies point to the relevance of the crucial period of hospitalization in rooming-in wards for the support and success of breastfeeding and that counseling is an effective Public Health intervention increasing the rates of breastfeeding, including the exclusive form, in different contexts and circumstances.

The study is feasible and safe, does not require invasive procedures, and does not interfere with the institutional care received by the participants.

Trained researchers will apply the intervention using a breastfeeding counseling approach, lasting approximately 30 min, depending on the participant’s demands.

The research is relevant because, so far, no clinical, randomized, and national multicenter trial has been identified that studied the effectiveness of individualized counseling carried out by a trained nurse, carried out during hospitalization in rooming-in wards, during the duration of exclusive breastfeeding.

In addition, it is noteworthy that although studies prove the effectiveness of counseling on breastfeeding, no studies were identified that carried out the intervention only during hospitalization in rooming-in wards. A meta-analysis on breastfeeding counseling identified that the intervention was applied during pregnancy or home visits after birth. With this, this study aims to deepen discussions about the length of stay of the dyad in the rooming-in wards as an opportunity to promote breastfeeding.

Thus, it is expected that the results of this clinical trial provide evidence that contributes to supporting women and increasing the duration of exclusive breastfeeding, precisely, the counseling approach developed by nurses during the hospitalization of the dyad in rooming-in wards.

### Clinical trial status

The study protocol’s final version (version 1) occurred on January 9, 2022. Recruitment of participants for the pilot study began on March 1, 2023, and is estimated to be completed by July, 2023. The clinical trial is expected to start collection in November 2023 and estimate the end of recruitment in January 2024 and completion of the study in May 2024.

### Supplementary Information

**Additional file 1.**

## Data Availability

Any data required to support the protocol can be supplied on request.

## Appendix

### INFORMED CONSENT FORM (ICF)

The objective of this research is to verify the effectiveness, that is, the results, of counseling, which will be individualized (for each person) and carried out by a nurse who has been trained in this skill, during hospitalization in the Rooming-in (ward where mother and child stay together after delivery). These results will be evaluated according to the maintenance of exclusive breastfeeding until the child's sixth month. They will be compared with usual care (provided by professionals from the institution).

We want to count on your participation because the weaning rate in Brazilian children is very high; previous studies show how important this period of hospitalization in Rooming-in is to obtain support and success in breastfeeding; this counseling approach has been previously tested and is an effective Public Health intervention, that is, it has positive results in maintaining exclusive breastfeeding (only breast milk offered to the baby). However, despite these benefits, no study has been carried out to date that has tested this approach during hospitalization in Rooming-in, nor has any national study involving several maternity hospitals been carried out, as is the case of this study. We emphasize that this study will be carried out at the same time in the rooming units of the Clinics Hospital of the Federal University of Triangle Mineiro, in Uberaba, in the Maternidade Escola of the Federal University of Rio de Janeiro, in Rio de Janeiro, and also in the Hospital Inacia Pinto dos Santos, in Feira from Santana, Bahia. These are the reasons that justify the realization and importance of this study.

Accepting to participate in this research, it will be necessary to answer the interview data and the researcher questions and allow the researchers to evaluate the breasts and breastfeeding during your hospitalization. From the telephone number provided during the initial contact, the researchers will contact you in the first and second weeks after birth and in the first, fourth, and sixth months after discharge to determine how the baby is breastfeeding, facilities, and difficulties in this period.

Three contact attempts will be made from the number provided and an attempt to contact a family member. If we get this contact, we will be able to have the necessary follow-up of the research, and in this case, the follow-up will be realized.

The initial contact will be made in the Rooming-in ward without needing travel and will not interfere with routine hospital care. The interviews will be conducted by a duly trained member of the research team, with an estimated time of 30 min, with breastfeeding being evaluated twice a day until hospital discharge.

It is essential to highlight that there will be two study groups. This means there will be an independent drawing by a system external to the institution. Then you will have equal chances to join one group or the other. In a group, the intervention will be carried out, that is, the counseling approach, individually by a trained nurse. This will be our intervention group. If you are drawn into the control group, you must answer the interview, and your breastfeeding may be evaluated. However, the researcher will not intervene so that you will receive the institution's routine care. During the calls, there will be no intervention; they will only be to check your health conditions, your baby, and how breastfeeding is going. Follow-up will follow the same protocol for both groups.

The expected risks of your participation in this research are loss of secrecy and privacy and damage to hospital routines. As measures to minimize these risks, the following measures will be taken: the interviews will be identified by numerical codes, and before approaching the participants, the care team will be asked about the best time to carry out the research. It will be informed that the participant will be participating in the research and where it is located to ensure continuity of care and planned care.

As a direct benefit of their participation in the research: the two groups will receive educational material as a video scribe on the main doubts and complications with breastfeeding after discharge. During telephone contact, they will be guided to seek support when the need is detected. Thus, both groups will benefit, directly or indirectly, through health education and post-discharge contact with a qualified professional.

Your participation is voluntary, and as a result, you will not receive any cash value. You will not incur any costs for participating in this study, as any costs you incur due to this research will be reimbursed.

You can refuse to participate in the study or withdraw at any time without prejudice to the assistance you are receiving. At any time, you can obtain any information about your participation in this research directly from the researchers or by contacting the Research Ethics Committee of the Clinics Hospital of the Federal University of Triangle Mineiro.

Your identity will not be revealed to anyone; it will be known only to the research researchers; your data will be published together without the risk of being identified, maintaining your secrecy and privacy. You can claim compensation for any damages you suffer due to this research.

The data obtained from you, collected in forms, will only be used for this research and destroyed and discarded five years after the end of the research. Suppose the researchers are interested in using your data in another research project. In that case, you will be contacted again to decide whether or not to participate in this new research, and if you agree, you must sign a new Informed Consent Form.

Data from the study coordinator and the Research Ethics Committee.

I, ____________________________________________________________, have read or heard the above clarification regarding the research “Effectiveness of individualized counseling on the duration of exclusive breastfeeding: a multicenter, randomized, parallel and open clinical trial.” I understand what the research is for and what procedures I will be submitting. The explanation I received clarifies the risks and benefits of the research. I understand that I am free to discontinue my participation at any time without justifying my decision and that this will not affect the assistance I am receiving. I know that my name will not be disclosed, I will not incur expenses, and I will not receive money to participate in the research. I agree to participate in the research, “Effectiveness of individualized counseling on the duration of exclusive breastfeeding: multicenter, randomized, parallel and open clinical trial,” I will receive a signed copy of this document.

Signatures: coordinator, researcher and participant.

## Abbreviations

BA: Bahia (Brazilian state)
FDA: Food and Drug Administration
HC-UFTM: Clinics of the Federal University of Triangle Mineiro
HIPS: Hospital Inacia Pinto dos Santos
LATCH: Validated scale that evaluates: latching, swallowing, nipple type, comfort and positioning
MG: Minas Gerais (Brazilian state)
NIH: National Institutes of Health
ReBEC: Brazilian Registry of Clinical Trials
RCT: Randomized controlled trial
RJ: Rio de Janeiro (Brazilian state)
RR: Relative risk
SPIRIT: Standard Protocol Items: Recommendations for Interventional Trials
SPSS: Statistical Package for the Social Sciences
SUS: Health Unic System
